# Improving Nurse Participation in Family-Centered Rounds in General Pediatrics: A Quality Improvement Initiative

**DOI:** 10.7759/cureus.106927

**Published:** 2026-04-12

**Authors:** Aza S AlSawafi, Murtadha M Al Lawati, Kadhiya N Al Azri, Athar H Al Riyami, Anoud A AlSaidi, Suhair K AlAbri

**Affiliations:** 1 Pediatric Medicine, The Royal Hospital, Muscat, OMN; 2 Pediatrics, Medical City for Military and Security Services, Muscat, OMN; 3 Pediatrics, Samail Hospital, Samail, OMN; 4 Pediatrics, Sohar Hospital, Sohar, OMN; 5 Nursing, The Royal Hospital, Muscat, OMN

**Keywords:** family-centered rounds, general ward, nurse-physician communication, pediatric, quality improvement projects

## Abstract

Introduction and background

Family-centered rounds (FCRs) are the cornerstone of collaborative pediatric inpatient care, promoting communication and shared decision-making among healthcare teams and families. However, active participation by assigned nurses is often inconsistent, which may limit optimal care coordination.

Objective

This quality improvement (QI) project aimed to enhance assigned nurse attendance during physician-led FCRs of the general pediatric team at a tertiary hospital.

Methods

A multidisciplinary team utilized a driver diagram to identify key barriers to nurse participation, including scheduling conflicts, unclear expectations, and workflow disruptions. Targeted interventions were introduced over a 10-week period. These included the implementation of a dedicated Doctors-Nurses schedule (DNS), a registrar checklist tool, and recognition strategies to reinforce engagement. Key metrics tracked included nurse attendance, nurses’ execution of daily treatment plans, checklist tool compliance, and FCR duration.

Results

Nurse attendance rose from a baseline level of 0% to 91.3% and 94% during the first two Plan-Do-Study-Act (PDSA) cycles, ultimately reaching 100% by the 10th week. Among participating nurses, 80.8% consistently and completely executed assigned treatment plans. Checklist tool compliance remained high at 100% throughout the project. Importantly, there was no statistically significant change in the average duration of rounds (p-value = 0.529), suggesting that the improvements did not negatively impact workflow efficiency.

Conclusion

Implementing structured, team-led interventions significantly improved nurse engagement in FCRs. This enhanced interdisciplinary collaboration and communication, contributing to efficient and more effective pediatric inpatient care without prolonging clinical workflow.

## Introduction

Ward rounds are a core clinical practice in which healthcare professionals collaborate to review inpatient care, share information, and plan management. In pediatric settings, they are essential for delivering coordinated, patient- and family-centered care (PFCC) [1,2]. To enhance collaboration and actively involve patients and families, traditional ward rounds have evolved into structured family-centered rounds (FCRs).

FCRs are a structured approach to inpatient care in which patients and their families actively participate in clinical discussions and decision-making alongside the interdisciplinary healthcare team [1,2]. This model is grounded in the principles of PFCC, emphasizing collaboration, communication, and shared decision-making to improve care quality and patient outcomes [1,2]. Active participation of all team members, including nurses, is essential to fully realize the benefits of this approach.

Growing evidence highlights the positive impact of nurse participation in ward rounds. Their presence enhances shared decision-making, medication safety, discharge preparedness, and family satisfaction - key indicators of high-quality pediatric care [3,4]. Additionally, nurse involvement strengthens communication, fosters interdisciplinary collaboration, and contributes to improved clinical outcomes [5,6].

At our institution, however, nurses are often not present during physician-led FCRs in the general pediatric wards. This gap, influenced by operational and workflow challenges, may limit opportunities for integrated and holistic care, which is an essential principle in pediatrics.

To address this issue and promote the active involvement of nurses in FCRs, we launched a quality improvement (QI) project with the goal of enhancing their consistent presence and engagement.

## Materials and methods

Setting and context

This QI initiative was conducted over a 10-week period, from December 2023 to March 2024, in the general pediatric wards at the Royal Hospital, a tertiary care referral center located in Muscat, the capital of the Sultanate of Oman.

The general pediatric service at the Child Health Department consists of three general pediatric teams (Teams A, B, and C), each responsible for admitting and managing patients within the general pediatric wards. Each team typically comprises two to three junior clerkship students, three to four interns, three to four pediatric residents, and one general pediatric consultant. Clinical team composition is dynamic, with junior clerkship students, interns, and pediatric residents rotating every four weeks, while general pediatric consultants rotate between teams every two to four weeks.

Given this frequent turnover, which may impact continuity of care and the implementation of QI interventions, the project team elected to pilot the initiative within a single team. Accordingly, the initiative was implemented and led by General Pediatric Team B.

Problem description and aim

The aim of this QI initiative was to increase the attendance of assigned nurses during physician-led FCRs and promote their engagement in patient care discussions.

The project’s SMART aim was defined as follows: by the end of February 2024, at least 50% of children admitted under Team B at the Royal Hospital would have their assigned nurse present during physician-led FCRs.

Prior to the intervention, a brief survey was conducted among pediatric nurses working in the general pediatric wards to assess their willingness to participate in FCRs. The results demonstrated that over 80% of nurses expressed willingness to attend and engage in clinical rounds, indicating strong baseline support for their involvement.

Intervention development

Following the initial assessment, a multidisciplinary focus group was formed to explore barriers and facilitators to nurse participation in FCRs. This group consisted of four senior pediatric residents, five in-charge staff nurses, and one general pediatric consultant.

**Figure 1:**
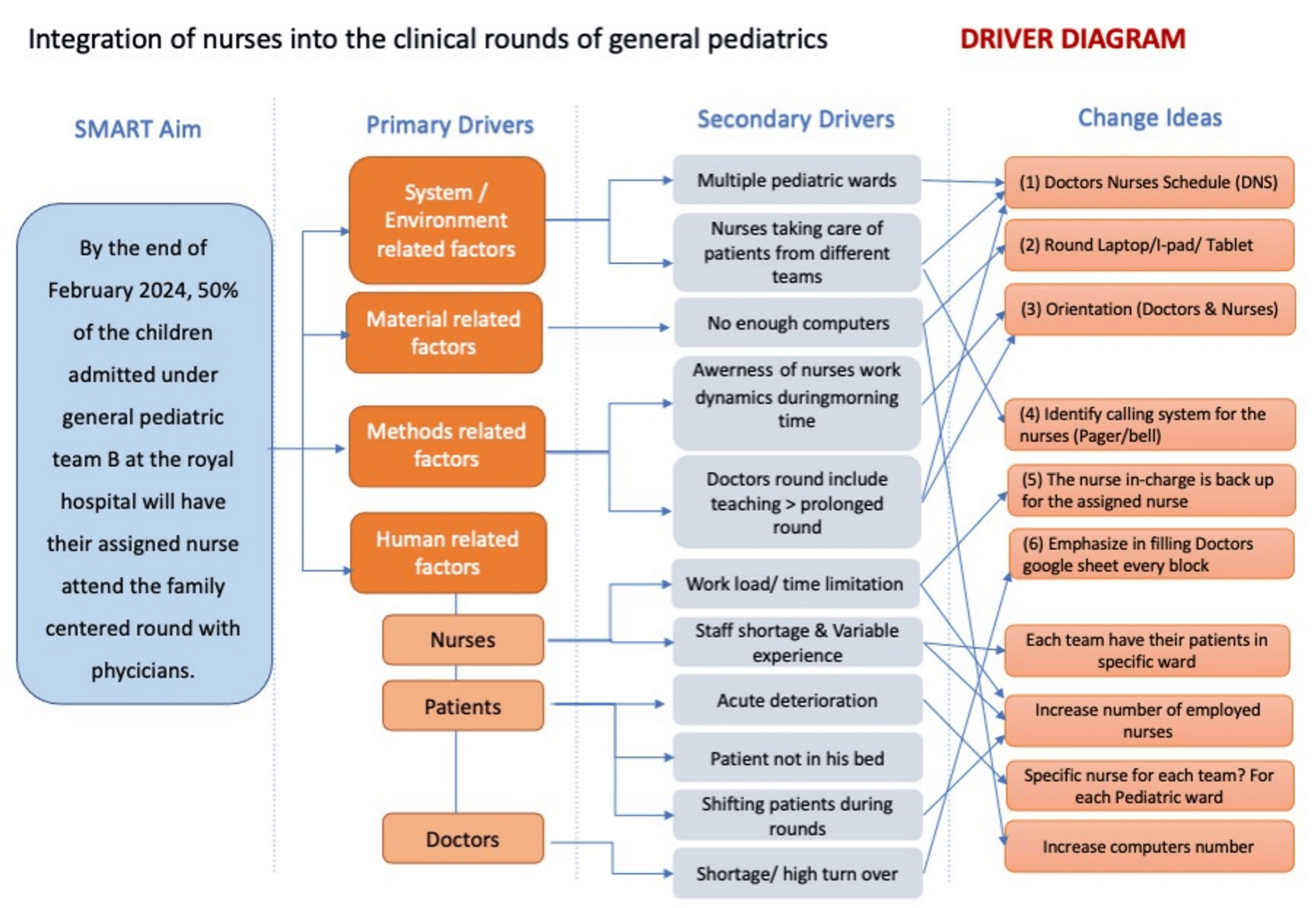
Driver diagram showing the primary and secondary drivers and the change ideas created by the quality improvement (QI) team during their brainstorming sessions.

The group conducted a structured brainstorming session during which a key driver diagram was developed to systematically identify factors influencing nurse attendance and engagement during FCRs (Figure 1). These included both barriers (e.g., workflow challenges and communication gaps) and facilitators (e.g., team coordination and role clarity). This process enabled the team to prioritize actionable areas for improvement and guided the development of targeted intervention strategies, which were then implemented as described below.

Interventions

Several targeted interventions were implemented to enhance nurse participation during FCRs. These interventions were designed based on the key driver diagram and implemented simultaneously.

At the start of each resident rotation (every four weeks), orientation presentations were conducted to familiarize new residents, registrars, and interns with the QI project, its objectives, and workflow expectations. The Doctors-Nurses Schedule (DNS; see Appendix 1) was completed each morning by the senior resident to document patient assignments and expected round timings, allowing real-time tracking of nurse attendance. The registrar checklist guided the registrar to follow the project plan and actively engage nurses during FCRs. Round laptops or tablets were provided to enable immediate access to electronic medical records and facilitate documentation during FCRs. A backup plan designated the ward-in-charge nurse to attend rounds when the assigned nurse was unavailable. Finally, positive reinforcement, in the form of daily verbal appreciation and recognition certificates, was provided to nurses who participated in FCRs, promoting engagement and adherence.

These interventions were continuously monitored and refined throughout the initiative using iterative Plan-Do-Study-Act (PDSA) cycles, ensuring that both adherence and effectiveness were optimized (Table 1).

**Table 1:** Summarizes the interventions, their descriptions, and the target groups.

Intervention	Description	Target Group/Focus
Doctors-Nurses Schedule (DNS)	Collaborative tool completed during the morning huddle to agree on assignments and expected round timings.	Doctors and nurses
Registrar Checklist	Structured guide for the registrar (specialist) to follow the project plan and prompt nurse participation in family-centered rounds (FCRs).	Registrar
Orientation Presentations	Conducted at the start of each resident rotation (every 4 weeks) to orient new residents to the quality improvement (QI) project and expectations.	New registrars, residents & interns
Round Laptop/Tablet	Provided real-time access to electronic medical records for immediate data entry and written communication during FCRs.	Entire clinical team
Backup Plan	Ward in-charge nurse designated as backup when the assigned nurse was unavailable.	Nurses
Positive Reinforcement	Recognition certificates and daily verbal appreciation for nurses participating in FCRs.	Nurses

Ethical consideration

This study was conducted as a QI initiative in accordance with local institutional policies. No patient-identifiable information was collected or disclosed, and all data were fully anonymized. Additionally, no personal or identifiable information of participating healthcare professionals (including physicians and nurses) was recorded.

As this project did not involve human subjects research and was limited to QI activities, institutional review board (IRB) approval was not required, and informed consent was also not required.

Study of the intervention and measures

The intervention was evaluated by prospectively tracking nurses’ attendance during physician-led FCRs on a daily basis. Attendance data were recorded using the DNS, which was completed by the senior resident of the team. For each patient, the DNS included the patient’s name, bed number, and the name of the assigned nurse. During the FCR, the senior resident indicated whether the assigned nurse attended the round.

At the end of each day, the QI team collected the completed DNS forms and entered the data into a structured electronic data collection form (Google Forms; Google, Inc., Mountain View, CA, USA) for analysis. Alongside nurse attendance, the team monitored additional process measures, including documentation of checklist utilization by the pediatric registrar during rounds and the duration of daily rounds over the 10-week initiative period.

Adherence to the planned interventions was also tracked by monitoring the execution of treatment plans by the assigned nurse. A designated senior nurse supervised this process and documented compliance on a daily basis, ensuring accurate capture of both outcome and process indicators.

The project team held regular meetings to review the collected data, assess progress, and implement refinements through iterative PDSA cycles every two to four weeks.

Categorical variables, including nurses’ attendance, were summarized as frequencies and percentages. Continuous variables, such as round duration, were summarized as mean ± standard deviation (SD) or median (interquartile range (IQR)), depending on data distribution. Comparisons of continuous variables between groups were performed using the independent t-test or Mann-Whitney U test, as appropriate. All analyses were conducted using Jamovi (The Jamovi Project, Sydney, Australia).

## Results

A total of 276 patient-nurse encounters were observed during physician-led FCRs throughout the study period. Each encounter represented a single patient seen by the assigned nurse during a rounding session. These encounters served as the sample for evaluating nurse attendance, adherence to the registrar checklist, and the execution of treatment plans.

Outcome measures

**Figure 2:**
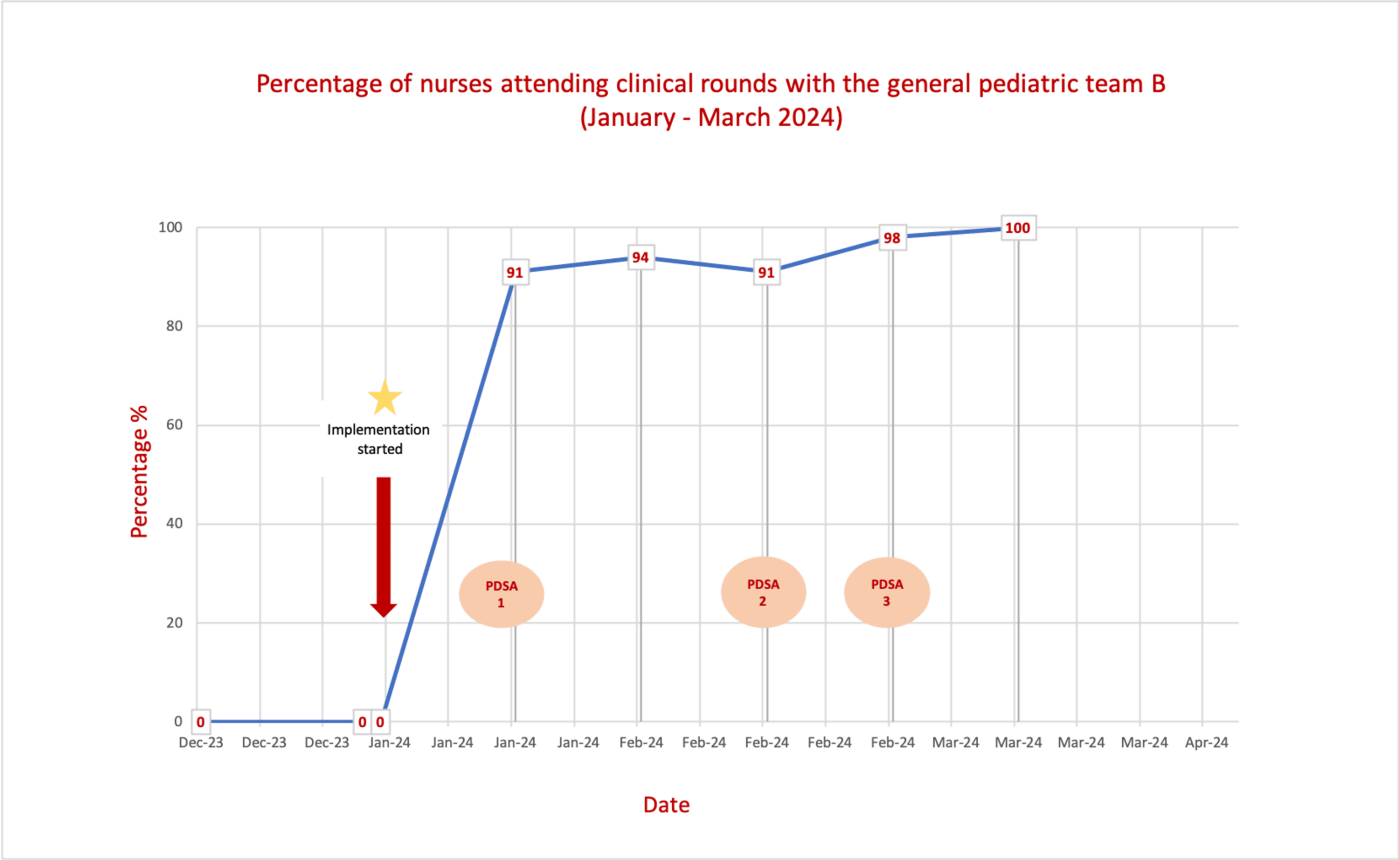
Run chart showing the percentage of nurses attending the clinical rounds of the General Pediatric Team B over the quality improvement (QI) time period. PDSA: Plan-Do-Study-Act

Prior to this QI project implementation, assigned nurses were not participating in the FCRs, with an attendance rate of 0%. However, following the QI interventions, the nurses’ attendance rate improved to 91.3% in the first PDSA cycle and 94% in the second. Furthermore, the nurses’ attendance rate was sustained at 100% over the final weeks of the project (Figure 2). With daily monitoring of the nurses participating in the FCRs with Team B by a dedicated senior nurse, we found that 80.8% of the nurses fully implemented treatment plans, 18.5% partially implemented them, and only 1.2% did not complete implementation during their shifts.

Process measures

The registrar checklist, a key process tool designed to prompt nurse engagement during physician-led FCRs, demonstrated high adherence throughout the study period. Completion of the checklist was achieved in 100% of FCRs conducted by Team B. The checklist was completed by the pediatric registrar during each round and submitted to the QI team on a daily basis, allowing for continuous monitoring and documentation of process adherence.

This high completion rate was supported by multiple reinforcement strategies. Visual reminders, including posters placed in the main handover room, were used to prompt completion of the checklist as part of the routine workflow. In addition, daily engagement with nursing staff through regular meetings helped reinforce the importance of the intervention and maintain team awareness and accountability. These combined approaches facilitated the successful integration of the checklist into daily practice.

Balancing measures

To assess the potential impact of the intervention on workflow efficiency, the duration of daily rounds was monitored as a balancing measure. There was no statistically significant difference in the mean round duration between Team B and one of the other general pediatric teams (120 ± 24.5 minutes vs. 113 ± 27.6 minutes; p = 0.529).

These findings suggest that the implementation of the QI interventions did not result in a meaningful increase in rounding time or any disruption to clinical workflow.

## Discussion

This QI initiative demonstrated that the implementation of structured, multidisciplinary interventions can significantly enhance nurse participation in physician-led FCRs. The consistent use of key process tools, particularly the DNS and the registrar checklist, enabled the team to achieve and sustain full participation of assigned nurses, with attendance rates reaching 100% during the study period. This improvement was associated with better care coordination and timely execution of treatment plans, reflecting enhanced interdisciplinary collaboration, which has been widely recognized as a cornerstone of PFCC [2].

In line with these findings, previous QI initiatives have reported similar improvements in nurse participation. Sharma et al. observed an increase in nursing attendance during FCRs from 47% to 80% following the introduction of structured communication strategies [7]. Similarly, Johnson et al. demonstrated improvement in nurse attendance from 33% to 100% through barrier identification, implementation of structured frameworks, and ongoing audit and feedback processes [8]. More broadly, structured interventions aimed at improving interdisciplinary communication and workflow have been shown to enhance nurse engagement in bedside rounds and improve care processes and outcomes [4,9-12].

Beyond consistency with prior studies, the significance of these findings lies in their impact on care delivery. Effective interdisciplinary collaboration has been associated with enhanced patient-centered care, improved communication, and better clinical outcomes [10,13]. Evidence from systematic reviews suggests that structured interdisciplinary bedside rounds improve patient-centeredness, quality of care, and team collaboration when implemented effectively [13]. Additionally, increased nurse involvement supports professional autonomy and accountability in clinical decision-making, further strengthening care delivery [3].

Importantly, in our study, this improved participation translated into measurable process benefits. Increased nurse involvement in FCRs led to better adherence to management plans, as observed through daily monitoring. This highlights the practical impact of interdisciplinary collaboration beyond attendance alone. Previous studies have demonstrated that active nurse participation in clinical rounds can improve patient outcomes, including reductions in hospital length of stay and improvements in patient satisfaction scores [12]. Furthermore, improving nurse-physician communication has been shown to enhance patient experience and care coordination in hospital settings [6].

The success of this initiative can be attributed to several key implementation strategies. A major strength was the structured and theory-informed development of interventions. Early engagement of frontline staff, particularly nurses, through a multidisciplinary focus group and key driver diagram exercise allowed identification of context-specific barriers and facilitators. This participatory approach ensured that the interventions were practical, acceptable, and feasible within the local clinical environment, consistent with best practices in QI methodology [1,11]. The use of multiple complementary strategies - such as orientation sessions, structured tools, workflow adjustments, and positive reinforcement - likely contributed to the high level of adherence and sustainability observed [4,7].

In addition to improving participation, it was important to ensure that these interventions did not negatively affect workflow efficiency. The duration of FCRs remained comparable to other teams, with no statistically significant increase in rounding time. This finding aligns with previous evidence indicating that structured, schedule-based FCRs can improve nurse attendance, enhance family engagement, and maintain provider satisfaction without compromising efficiency or educational value [4,5].

Despite these positive findings, several limitations should be considered when interpreting the results. First, the study was conducted within a single pediatric team in one tertiary care center, which may limit generalizability. Second, nurse attendance and checklist completion were based on daily documentation by team members, which may introduce reporting bias. Third, while process measures and nurse participation improved, the study did not assess direct patient-centered outcomes, such as caregiver satisfaction, patient experience, or clinical outcomes (e.g., length of stay). Additionally, the presence of the QI team and ongoing monitoring may have influenced behavior (Hawthorne effect), potentially overestimating adherence during the study period [11].

Building on these findings, consideration of long-term sustainability is essential. Ensuring sustained improvement will require embedding these interventions into routine practice. Strategies include integrating the DNS and registrar checklist into standard operating procedures, providing orientation for new residents and nurses at the start of each rotation, and conducting periodic audits with structured feedback to monitor adherence. Leadership endorsement and continuous engagement of frontline staff will be essential to maintain performance despite staff turnover or leadership changes. Establishing a culture of interdisciplinary collaboration and accountability can further promote sustained improvement [1,4,11].

Finally, further research is needed to expand on these findings. Future work should focus on scaling this intervention across multiple teams and evaluating its impact on patient-centered and clinical outcomes. Incorporating patient and family perspectives, as well as assessing sustainability over longer periods, would further strengthen the evidence base. Additionally, adapting the intervention to different clinical contexts may require consideration of local workflow dynamics and resource availability, as highlighted in previous implementation studies [1,4].

## Conclusions

This QI initiative demonstrated that structured, multidisciplinary interventions can effectively enhance nurse participation in physician-led FCRs. Orientation sessions, the DNS, registrar checklists, and positive reinforcement enabled Team B to achieve and sustain full nurse attendance. Increased participation contributed to better care coordination, timely execution of treatment plans, and strengthened interdisciplinary collaboration, all without prolonging rounding time or disrupting workflow.

Sustainability can be supported through embedding interventions into routine practice, providing orientation for new staff, and implementing periodic audits with feedback. Although limited to a single team, these strategies are practical, feasible, and have the potential for broader implementation. Future studies should evaluate the impact on patient-centered outcomes, clinical efficiency, and long-term sustainability across multiple teams and clinical settings.

## Appendices

Appendix 1

**Table 2:** Doctors-Nurses Schedule (DNS)

Doctors-Nurses Schedule (DNS)					
For General Pediatric Team B Clinical Rounds					
						Date:					
Time	Ward	Bed	Patient Name	Assigned Nurse name	If Done, put ✓
						09:45-10:00	Team Teaching round				
10:00-10:10					
10:10-10:20					
10:20-10:30					
10:30-10:45	Team Teaching round				
10:45-11:00					
11:00 -11:10					
11:10-11:20					
11:20-11:30	Team Teaching round				
11:30-11:40					
11:40-11:50					
11:50-12:00					
12:00-12:10	Team Teaching round				
12:10-12:20					
12:20-12:30					
12:30-12:40					
12:40-12:50	Team Teaching round				
12:50-13:00					
13:00-13:10					
13:10-13:20					
The clinical round today ended at:					
